# The REMBRANDT study, a large collection of genomic data from brain cancer patients

**DOI:** 10.1038/sdata.2018.158

**Published:** 2018-08-14

**Authors:** Yuriy Gusev, Krithika Bhuvaneshwar, Lei Song, Jean-Claude Zenklusen, Howard Fine, Subha Madhavan

**Affiliations:** 1 Innovation Center for Biomedical Informatics (ICBI), Georgetown University Medical Center, Washington DC 20007, USA; 2 National Cancer Institute, Rockville, MD, USA; 3 NewYork Presbyterian Hospital, New York, NY, USA; 4 Cornell Medical School, New York, NY, USA

**Keywords:** Microarray analysis, Cancer genomics, Data publication and archiving, CNS cancer

## Abstract

The Rembrandt brain cancer dataset includes 671 patients collected from 14 contributing institutions from 2004–2006. It is accessible for conducting clinical translational research using the open access Georgetown Database of Cancer (G-DOC) platform. In addition, the raw and processed genomics and transcriptomics data have also been made available via the public NCBI GEO repository as a super series GSE108476. Such combined datasets would provide researchers with a unique opportunity to conduct integrative analysis of gene expression and copy number changes in patients alongside clinical outcomes (overall survival) using this large brain cancer study.

## Background & Summary

In 2005, cancer became one of the leading causes of mortality in the United States. At the time, new and innovative initiatives of molecular characterization were developed in an effort to break down the barriers of insufficient and incomplete data, especially for novel clinical research hypothesis generation and testing. Consistent characterization of genomic and molecular data in conjunction with clinical data is needed to improve prognosis for patients with similar molecular profiles. One such initiative was the Rembrandt project (REpository for Molecular BRAin Neoplasia DaTa), a joint initiative of NIH’s National Cancer Institute (NCI) and National Institute of Neurological Disorders and Stroke (NINDS). This project consisted of a large brain cancer patient-derived dataset that contained clinically annotated data generated through the Glioma Molecular Diagnostic Initiative (GDMI) from 874 glioma specimens comprising 566 gene expression arrays, 834 copy number arrays, and 13,472 clinical phenotype data points.

The consistent molecular characterization allowed the data to be analyzed, integrated, and redistributed through a web based online platform of the same name - REMBRANDT, hosted at the NCI. This publicly available online platform was built on novel biomedical infrastructure and allowed analysis of genetic data in conjunction with clinical data and was one of the earliest initiatives aimed at precision oncology. This project won the Service to America Award in 2005 (https://servicetoamericamedals.org/honorees/view_profile.php?profile=109). Madhavan *et al*^1^ demonstrated the power of the data portal through several case studies.

In 2015, the NCI retired the REMBRANDT data portal, and all molecular data including microarray gene expression, copy number, and clinical data were migrated to the Georgetown Database of Cancer (G-DOC)^2^. G-DOC makes available clinical and biospecimen data from this study for 671 patients through its public web portal.

**Figure 1:**
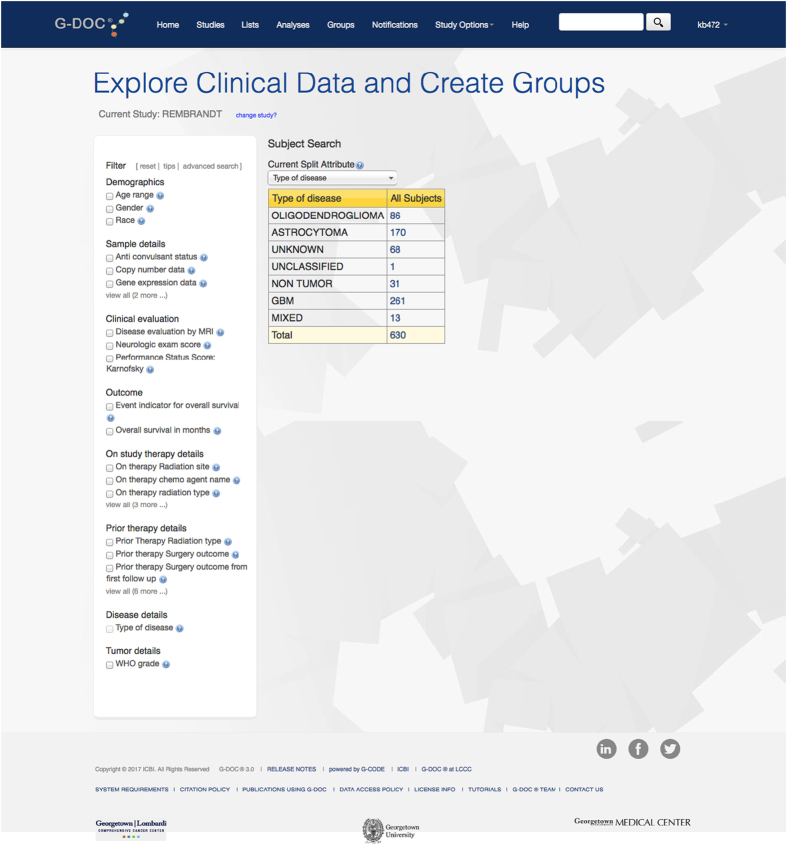
Screen shot of the Rembrandt dataset in G-DOC.

**Figure 2:**
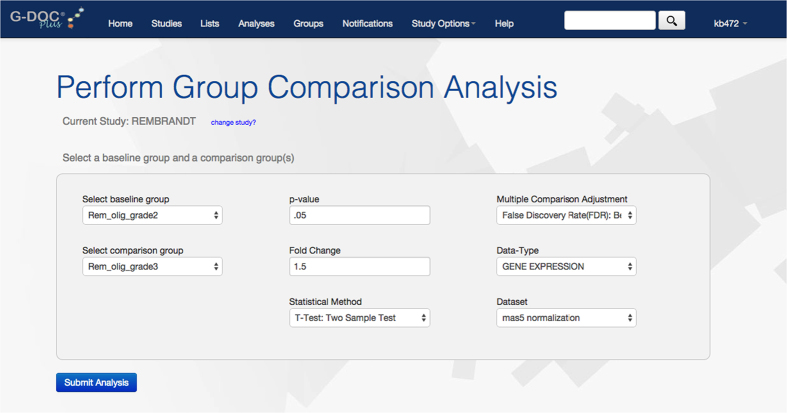
A screen shot from G-DOC showing the comparison of two groups of patients in the Rembrandt study–Oligodendroglioma patients with Grade II tumor and Oligodendroglioma patients with Grade III tumor.

G-DOC is a data integration platform that offers advanced computational tools to handle a variety of biomedical BIG DATA including gene expression arrays, next generation sequencing (NGS), metabolomics and medical images so that they can be analyzed in the full context of other omics and clinical information^2,3^ (Figs 1 and 2). After migration of the REMBRANDT dataset into G-DOC, we applied a novel algorithm for summarizing copy number data at the chromosome level called the Chromosomal Instability Index (CINdex), published as an open source BioConductor package (http://bioconductor.org/packages/CINdex/)^4^.

To augment the larger REMBRANDT project, a companion image collection was created that contained pre-surgical magnetic resonance images from 130 patients from the same REMBRANDT dataset, linked by Sample id to the G-DOC collection of clinical and molecular data. This image collection is now hosted at the Cancer Imaging Archive (TCIA) and available for public access (https://wiki.cancerimagingarchive.net/display/Public/REMBRANDT).

We believe that it would be a great service to the scientific community to make the REMBRANDT dataset available to the research community i.e. gene expression and matching copy number data from patients with brain cancers - both at segment level and processed CINdex level data along with de-identified clinical annotation including overall survival data. Such combined datasets would provide researchers with a unique opportunity to ask interesting questions of the molecular anomalies and correlate them to outcomes with the goal of generating novel testable hypothesis for biomarker development to treat patients diagnosed with Gliomas.

In this paper, we describe the REMBRANDT data set, sampling methodology, data processing methods that were applied and the online data platform that provides access to this data collection. We also describe how it can be accessed from other public data repositories.

All the raw and processed gene expression, copy number and the clinical data used for Rembrandt within G-DOC have been made public as a super series at the NCBI GEO repository (Data Citation 1).

## Methods

### Tissue samples

The NCI Neurooncology branch obtained an IRB approval from the NIH Clinical Center in 2003 to collect this dataset. Informed consent was obtained from all subjects. Matched tumor, blood, and plasma were collected from 14 contributing institutions including the NIH Clinical Center, Henry Ford Hospital, Thomas Jefferson University, University of California at San Francisco, H. Lee Moffitt Hospital, University of Wisconsin, University of Pittsburgh Medical Center, University of California at Los Angeles, The University of Texas M. D. Anderson Cancer Center, Dana-Farber Cancer Center, Duke University, Johns Hopkins University, Massachusetts General Hospital, and Memorial Sloan Kettering Cancer Center^1^. The dataset was fully deidentified to remove all HIPAA identifiers.

### RNA samples

Total RNA was extracted from the tumor tissue (50–80 mg) using the Trizol reagent (Invitrogen) and following the manufacturer’s instructions. The quality of RNA extracted was verified using Agilent’s Bioanalyzer System with the help of RNA Pico Chips. 5 μg RNA extracted from each sample was processed using the Affymetrix U133 Plus2 gene expression microarray chips^1^.

### Gene expression data pre-processing, quality control, and expression data normalization

The raw data files from all Affymetrix arrays that passed the minimal quality-control were normalized using the package (http://www.dchip.org/). The model-based expression index algorithm was applied (dChip). This algorithm selects an invariant set with a small within-subset rank difference to serve as the basis for adjusting brightness of the arrays to a comparable level. The normalization was done at the perfect match (PM) and mismatch (MM) probe levels, and model-based expression levels were calculated using normalized probe level data. The average difference model (PM > MM) was chosen to compute expression values; negative average differences were truncated to 1 or log-transformed values of zeros to flag negative signal intensities.

### Expression data pre-processing

For pre-processing, probe-level data were processed with custom Chip Definition Files that rearranged Affymetrix probes into gene-based probe sets. Probes mapped to alternatively spliced exons were grouped into distinct probe sets. Most 3′ probes were selected for processing. Nonspecific probes were masked before processing. Probe-level data were consolidated into probe-set data using the Affymetrix MAS5 algorithm, with the target scaling value at 500.

### DNA samples

Tissue (∼10 μg; as recommended by the manufacturer) from each tumor was used to extract high molecular weight, genomic DNA using QIAamp DNA Micro DNA extraction kit (Qiagen) following the manufacturer’s instructions. The quality of DNA was checked by electrophoresis run in a 2% agarose gel. Genomic DNA (250 ng) from samples received were hybridized to 100 K single nucleotide polymorphism chips (http://www.affymetrix.com/support/technical/byproduct.affx?product=100k), which covered 116,204 single nucleotide polymorphism loci in the human genome with a mean intermarker distance of 23.6 kb. These arrays give two simultaneous data types: allelic calls and signal intensity, allowing for the determination of both copy number alterations and regions of allelic imbalances (loss of heterozygosity).

### DNA Data Processing

Calls were determined by the GTYPE software (Affymetrix Inc, Santa Clara) version 3.0 with 25% level of confidence. Only samples with call rates of > 90% were accepted for any analysis. The 100 K arrays were a set of 2 chips, 50 K each, designed for different restriction enzyme - XbaI and HindIII. So each sample was analyzed on 2 arrays. These arrays were processed separately and looked for their concordance with HapMap data as described in Matsuzaki *et al*^5^. Genotyping performance was assessed by comparing subsets of genotypes with calls determined by sequencing and, most importantly, using concordance measure with data from the HapMap Project as described in Matsuzaki *et al*^5^. By tuning the cutoff filter, one can strike an optimal balance between call reliability and call rates for any given study. The recommended cutoff of 0.25 was applied and provided the concordance values above 99.5% for both arrays. In addition, the 100 K arrays platform provided built-in controls to cross-check for consistency of results between the arrays, Thirty-one SNPs on both the XbaI and HindIII arrays serve as built-in controls forthe array set. These controls allow researchers to cross-check genotypes from the same sample on each array to verify that both arrays remain together through array preparation protocols and data analysis, as described in this Affymetrix datasheet (http://tools.thermofisher.com/content/sfs/brochures/100k_datasheet.pdf).

### Data processing for G-DOC

We obtained the Rembrandt data collection from the NCI for loading to G-DOC. First, the pre-processed data were checked for integrity so that every patient had one matching clinical metadata, gene expression data array and (or) copy number data sample. While the gene expression data was already pre-processed, we applied our unique algorithm for copy number data analysis called Chromosomal Instability Index (CINdex). CINdex is available to the public as a BioConductor Package: http://bioconductor.org/packages/CINdex/^4^.

The CINdex package uses the segment level data to calculate the genomic instability in terms of copy number gains and losses separately at the chromosome and cytoband level. The genomic instability across a chromosome offers a global view (referred to as Chromosome CIN), and the genomic instability across cytobands regions provides higher resolution (referred to as Cytobands CIN) view of instability. This allows assessing the impacts of copy number alternations on various biological events or clinical outcomes by studying the association of CIN indices with those events.

The CINdex algorithm was applied on both the XbaI and HindIII Rembrandt copy number arrays, and made available through our GEO submission. The segment level information was obtained from the copy number array data in the.CN4.cnchp files, and input into the CINdex algorithm.

The Rembrandt dataset in G-DOC is summarized in Table 1. The Rembrandt clinical data in G-DOC (summarized in Table 2) had a total of 28 clinical attributes, which includes demographics, primary diagnosis, tumor stage and race. The complete set of clinical attributes including survival is provided in a comprehensive table as part of our GEO submission (Data Citation 1). The clinical data was checked for integrity and then mapped to the existing data structures as a precursor to loading within the G-DOC database. Several files were created, each that described the clinical attributes with respect to their type and vocabulary. Special files were also created that described the mapping between the clinical and gene expression data; and clinical and copy number data. The summary, study characteristics, and contact information were captured in a separate file. Once all metadata files were created, loading scripts were used to import the data into the G-DOC database. Duplicate biospecimen samples were excluded from the G-DOC database^2^.

The G-DOC system^3^ uses the Oracle 11 g relational database and consists of 44 common tables. For each new study loaded, a separate schema is created consisting of a set of 12 study-specific tables. All processed data files pertaining to a particular study are loaded separately onto a computation-centric server designed to handle high-throughput data analysis^2^.

After data processing and cleaning, there were a total of 671 patients with clinical data, of which 541 had gene expression data, and 507 patients had undergone SNP chip profiling. 263 patients had information about segment level copy number data. 220 patients had both gene expression and copy number data. Out of the total number of biospecimen files received from NCI, there were a total of 550 gene expression .CEL files and 16 copy number .CEL files. The level 2 gene expression data included 550 CHP files (http://dept.stat.lsa.umich.edu/~kshedden/Courses/Stat545/Notes/AffxFileFormats/chp.html) that contained the probe set analysis results generated by the Affymetrix software. The level 2 copy number data included a total of 1,992 files, which consisted of 1,484 CHP files that contained genotype calls; and 508 CN4.cnchp files (https://www.affymetrix.com/support/developer/powertools/changelog/gcos-agcc/cnchp-lohchp.html) that included copy number results generated from the Affymetrix CN4 algorithm. Out of these 1,992 files, 1,010 were profiled using Xba array (747 CHP files and 263 CN4.cnchp files), and 982 profiled on Hind array (737 CHP files and 245 CN4.cnchp files).

### Case study using Rembrandt dataset in G-DOC

**Figure 3:**
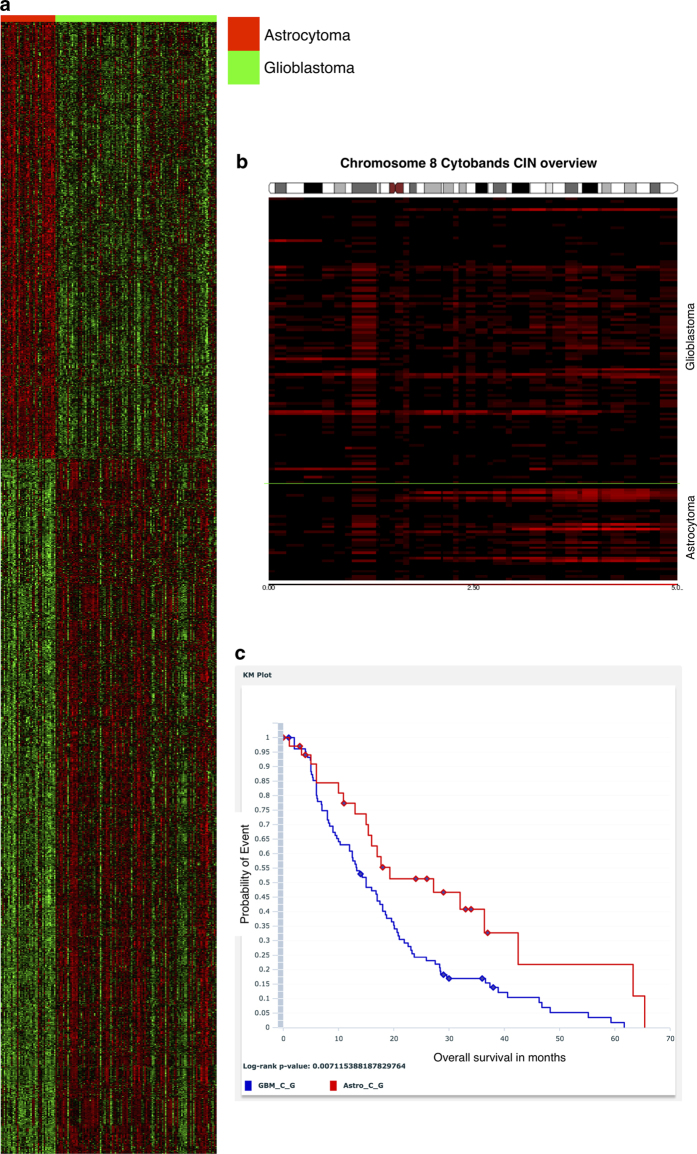
A case study comparing Astrocytoma and GBM patients using gene expression, copy number, and clinical data in the G-DOC platform. (**a**) Heat map comparing Astrocytoma and GBM patients. Over-expression of genes in the heat map is represented in red color, and under-expression is shown in green color. (**b**) Chromosome instability in chromosome 8. Here, black color indicates normal DNA copy number (i.e. no instability); and the red color indicates instability - higher the instability, the brighter the red color (**c**) Kaplan Meier survival plot between Astrocytoma (red line) and Glioblastoma patients (blue line)^3^.

Bhuvaneshwar et al details a case study comparing Astrocytoma (low grade glioma) patients with those afflicted with GBM (high grade glioma) from the Rembrandt dataset using the G-DOC platform^3^ (Fig. 3). The case study compared the two groups of patients using gene expression, Chromosomal Instability Index (CINdex) and overall survival. The most down-regulated gene RHOF was six fold under-expressed in the GBM group compared to the Astrocytoma group. This gene is known to be down regulated in GBM patients through the over expression of their activators^6^. From comparison of copy number data between the two glioma types we found a higher level of chromosomal instability in the Astrocytoma group in chromosome 8q arm (indicated by the bright red colors). Aberrations in the 8q arm in Astrocytoma patients are known in literature^7–9^ (Fig. 3b). Finally, the Kaplan Meier survival plot (Fig. 3c) feature in G-DOC Plus showed the expected result that patients with Astrocytoma had better survival rates than those with GBM with a p-value of less than 0.05 from log rank test. Such case studies show the power of these kinds of multi-omics data and analyses platforms, which allow users to generate new hypotheses by a click of buttons without performing any intensive data analyses of their own.

### Usability

The success of clinical research software applications such as G-DOC is dependent on understanding the complex cognitive processes of the intended user. However, despite recent IOM reports highlighting the significance of cognitive and human factors approaches for use in clinical research environments^10^, there is a paucity of research within this domain. We routinely apply human factors approaches^11^ to improve the user interfaces in G-DOC to improve the user experience. For example, the search interfaces follow the e-commerce shopping cart (Amazon, Zappos) like style sheets to allow users to easily select, filter and visualize datasets. User-selected analysis routines are moved to an asynchronous thread by the software application to allow users to use other features while the analysis is run in the background. They can then go to the analysis results page to view the results of analysis at a later time point. Such usability improvements have attracted over 4,200 users to the G-DOC system for translational research and training purposes.

### Relevance to TCGA cohort

The Cancer Genome Atlas (TCGA) is a comprehensive collection of multiple omics data from 33 different cancers. TCGA has two brain cancer dataset collections. One is a collection of 617 cases with grade IV gliomas referred to as TCGA-GBM (https://portal.gdc.cancer.gov/projects/TCGA-GBM)^12^. In 2015, TCGA included a cohort of lower grade glioma cases (TCGA-LGG) (https://portal.gdc.cancer.gov/projects/TCGA-LGG)^13^ that included 517 grades II and III brain cancer cases. In contrast, the Rembrandt dataset contains clinical and molecular data on 671 cases from grade II, III, and IV gliomas. The TCGA brain cancer collection was used to determine subtype classification of tumors based on multi-omics profiling of sampless^14,15^.

The REMBRANDT collection is a large single study collection of brain cancers that was developed independent of TCGA efforts and provides a unique independent validation dataset for comparative analysis with TCGA. For instance Cooper et al corroborated the subtype classification obtained from the TCGA data using the Rembrandt dataset^16^ (https://cancergenome.nih.gov/researchhighlights/researchbriefs/corroboratesubtypes). This dataset lends itself to development of additional machine learning approaches including deep learning methods for assessing clinical relevance of biomarkers for diagnostic or therapeutic development.

### Rembrandt is FAIR-Compliant

The Rembrandt dataset is compliant with FAIR (Findable, Accessible, Interoperable, and Re-usable) data principles. With respect to these standards, the Rembrandt dataset is ‘findable’–previously as a standalone portal, and now hosted in G-DOC, with provenance and raw data available in the National Institute of Health (NIH) Gene Expression Omnibus (GEO) data repository. All these resources mentioned are publicly available and hence satisfy the ‘accessible’ condition. The gene expression and copy number data are in standard data matrix (MAGE-TAB) formats that support formal sharing and satisfy the ‘interoperable’ condition. Finally, this dataset is easily ‘reusable’ for additional research through either the G-DOC platform, or via GEO (Data Citation 1).

### Code availability

CINdex package is available to the public as a BioConductor package: http://bioconductor.org/packages/CINdex/^4^.

## Data Records

The raw gene expression and copy number data are available in Gene Expression Omnibus (GEO) as a super series (Data Citation 1). The gene expression files include the raw files in the form of .CEL files; processed data in the form of .CHP files. The raw gene expression is also available at ArrayExpress (Data Citation 2).

The copy number data deposited in GEO includes raw .CEL files, and probe set analysis results generated from Affymetrix software in the form of CHP and CN4.cnchp files. In addition, chromosome instability information obtained from the CINdex package is also available in the form of data matrices.

## Technical Validation

Quality Control was conducted on all microarrays according to NCI internal standard operating procedures. All arrays were confirmed to be within acceptable minimal quality-control variables following these criteria: (a) A scaling factor of < 5 when the expression values are scaled to a target mean signal intensity of 500. (b) Signal intensity ratios of the 3′ to 5′ end of the internal control genes of β-actin and GAPDH < 3. (c) Affymetrix spike control (BioC, BioDN, and CreX) are always present, and percentage present calls is > 35% for brain tissue^1^.

## Usage notes

The Madhavan *et al*^1^ publication that described the Rembrandt portal and dataset has enabled numerous analyses and has been cited 233 times so far (as of April 2018).

We believe that by making this dataset available to the research community via a public analysis-ready platform like G-DOC, and access to raw data via a public repository like GEO provides a unique data science research opportunity to the biomedical and data science research communities. Such combined datasets would provide researchers with a unique opportunity to conduct integrative analysis of gene expression and copy number changes alongside clinical outcomes (overall survival) in this large brain cancer study published to date.

## Additional information

**How to cite this article**: Gusev, Y. *et al.* The REMBRANDT study, a large collection of genomic data from brain cancer patients. *Sci. Data* 5:180158 doi: 10.1038/sdata.2018.158 (2018).

**Publisher**’**s note**: Springer Nature remains neutral with regard to jurisdictional claims in published maps and institutional affiliations.

## Supplementary Material



## Figures and Tables

**Table 1:** Details of the REMBRANDT dataset in G-DOC.

Source	Protocol 1	Samples	Protocol 2	Data
Rembrandt glioma samples	RNA extraction	671 patients	Microarray hybridization	GSE108474
Rembrandt glioma samples	DNA extraction	263 patients	SNP array hybridization	GSE108475

**Table 2:** Summary of the Rembrandt dataset.

	Clinical Attribute	Number of patients	% Of patients
Gender	Male	326	48.6%
	Female	177	26.4%
	Blank/NA	168	25.0%
Disease Type	GBM	261	38.9%
	Astrocytoma	170	25.3%
	Oligodendroglioma	86	12.8%
	Non tumor	31	4.6%
	Unknown	68	10.1%
	Unclassified	1	0.1%
	Mixed	13	1.9%
	Blank/NA	41	6.1%
WHO Grade	I	2	0.3%
	II	110	16.4%
	III	93	13.9%
	IV	140	20.9%
	Blank/NA	326	48.6%
Race	White	433	64.5%
	Black	15	2.2%
	Asian	7	1.0%
	Hispanic	1	0.1%
	Native Hawaiian	3	0.4%
	Unknown	7	1.0%
	Other	5	0.7%
	Blank/NA	200	29.8%
